# Laboratory Prototype of Bioreactor for Oxidation of Toxic D-Lactate Using Yeast Cells Overproducing D-Lactate Cytochrome *c* Oxidoreductase

**DOI:** 10.1155/2016/4652876

**Published:** 2016-06-30

**Authors:** Maria Karkovska, Oleh Smutok, Mykhailo Gonchar

**Affiliations:** Department of Analytical Biotechnology, Institute of Cell Biology, Drahomanov Street 14/16, Lviv 79005, Ukraine

## Abstract

D-lactate is a natural component of many fermented foods like yogurts, sour milk, cheeses, and pickles vegetable products. D-lactate in high concentrations is toxic for children and people with short bowel syndrome and provokes encephalopathy. These facts convincingly demonstrate a need for effective tools for the D-lactate removal from some food products. The main idea of investigation is focused on application of recombinant thermotolerant methylotrophic yeast* Hansenula polymorpha* “tr6,” overproducing D-lactate: cytochrome* c* oxidoreductase (EC 1.1.2.4, D*-*lactate cytochrome* c* oxidoreductase, D-lactate dehydrogenase (cytochrome), DLDH). In addition to 6-fold overexpression of DLDH under a strong constitutive promoter (*prAOX*), the strain of* H. polymorpha* “tr6” (*gcr1 catX/Δcyb2*,* prAOX_DLDH*) is characterized by impairment in glucose repression of AOX promoter, devoid of catalase and L-lactate-cytochrome *c* oxidoreductase activities. Overexpression of DLDH coupling with the deletion of L-lactate-cytochrome *c* oxidoreductase activity opens possibility for usage of the strain as a base for construction of bioreactor for removing D-lactate from fermented products due to oxidation to nontoxic pyruvate. A laboratory prototype of column-type bioreactor for removing a toxic D-lactate from model solution based on permeabilized cells of the* H. polymorpha* “tr6” and alginate gel was constructed and efficiency of this process was tested.

## 1. Introduction

D-lactate is a natural component of many fermented foods like yogurts [1, 2], sour milk [3], cheeses [4], and pickles vegetable products [5].

D-lactate can be metabolized slowly in the human body in comparison with L-lactate isomer and can cause metabolic disorders if ingested in excess. Therefore, the World Health Organization (WHO) recommends a limited daily consumption of D-lactate up to 100 mg·kg^−1^ bodyweight. Moreover, some countries, for example, Germany, have decided to minimize the D-lactate content of fermented dairy products [6]. The high concentration of D-lactate is toxic for children [7] and people with short bowel syndrome [8–10]. Also D-lactate toxicity depends on physiological state of renal excretion. Hingorani et al. demonstrated that, in healthy men continuously infused with a D-lactate solution, excretion rates ranged between 61 and 100% [11]. In 1993, there was reported a case of chronic D-lactate encephalopathy occurring in a patient with short bowel syndrome and end-stage renal disease [12].

Originally, it was believed that, due to the lack of enzyme D-lactate dehydrogenase in humans, they are not able to metabolize D-lactate to pyruvate. However, in the past two decades there is an abundance of literature suggesting the presence of a D-lactate metabolizing enzyme D-2-hydroxy acid dehydrogenase (D-2-HDH) that is mainly found in the liver and kidney [10, 13]. This enzyme is inhibited by low-pH states, which assumes importance in the relative overproduction of D-lactate in certain clinical situations. D-lactic acidosis or D-lactate encephalopathy is a rare condition that occurs primarily in individuals who have a history of short bowel syndrome (SBS).

The predominant organ system affected by D-lactic acidosis is the central nervous system (CNS). Presenting symptoms may include slurred speech, ataxia, altered mental status, psychosis, or even coma [8, 9, 14, 15].

The aim of the current work is construction of an effective laboratory prototype of bioreactor for removing D-lactate from fermented products based on the use of yeast cells overproducing D-lactate oxidoreductase. The D-lactate oxidoreductase (cytochrome* c*-dependent dehydrogenase, DLDH, EC1.1.2.4) is a FAD- and Zn^2+^-containing membrane-associated protein found in yeast and bacteria. The enzyme catalyzes D-lactate oxidation to pyruvate coupled with ferricytochrome* c* reduction to ferrocytochrome *c*. It is characterized as a mitochondrial protein with a molecular weight of 63 kDa for DLDH from* Kluyveromyces lactis* and 64 kDa for DLDH from* Saccharomyces cerevisiae*, respectively [16, 17]. DLDH is highly selective to D-lactate; however, D,L-*α*-hydroxybutyric acid can be used as an alternative electron donor. The enzyme is not selective with respect to electron acceptors and can reduce, in addition to its native acceptor (cytochrome* c*), different low molecular artificial redox mediators (ferricyanide, dichlorphenol indophenols, etc.).

We propose using recombinant DLDH-overproducing strain* H. polymorpha* “tr6” (*gcr1 catX/Δcyb2*,* prAOX_DLDH*) as a base for construction of laboratory prototype of bioreactor for removing a toxic D-lactate from fermented products. The column-type bioreactor includes alginate gel formations with incorporated permeabilized yeast cells.

## 2. Material and Methods

### 2.1. Materials

Sodium D-lactate, sodium alginate, and phenylmethylsulfonyl fluoride (PMSF) were purchased from Sigma-Aldrich Corp. (Deisenhofen, Germany) and cetyltrimethylammonium bromide (CTAB) was purchased from Chemapol Sp. (Bratislava, Slovakia).* D*(+)-glucose monohydrate was purchased from J. T. Baker (Deventer, Netherlands). (NH_4_)_2_SO_4_, Na_2_HPO_4_, KН_2_РО_4_, MgSO_4_, and CaCl_2_ were obtained from Merck (Darmstadt, Germany). All chemicals and reagents were of analytical grade and all solutions were prepared using deionized water. D-lactate standard solution and appropriate dilutions were prepared in 100 mM phosphate buffer (PB), pH 7.8.

### 2.2. Cultivation and Permeabilization of Yeast Cells

Recombinant DLDH-overproducing strain* H. polymorpha* “tr6” (*gcr1 catX/Δcyb2*,* prAOX_DLDH*) is characterized by 6-fold overexpression of DLDH, impaired in glucose repression, devoid of catalase and specific L-lactate-cytochrome* c* oxidoreductase activities, which was constructed by us earlier [18].

Cultivation of the* H. polymorpha* “tr6” was performed in flasks on a shaker (200 rpm) at 28°C until the middle of the stationary growth phase (~52 h) in a medium containing (g·L^−1^): (NH_4_)_2_SO_4_, 3.5; KH_2_PO_4_, 1.0; and MgSO_4_  ×  7H_2_O, 0.5, supplemented with 0.75% yeast extract. A mixture of glucose (10 g·L^−1^) and D-lactate (2 g·L^−1^) was used as a carbon and energy source and (1 g·L^−1^) methanol as an inductor for* AOX* promoter. After washing, the cells were suspended in 50 mM PB, pH 7.8, containing 1 mM PMSF.

Before experiments, the freshly prepared yeast cells were resuspended to 30 mg·mL^−1^ in 50 mM PB, pH 7.8, and stored in refrigerator.

The procedure of the cells permeabilization was as follows: the same volume of permeabilizing reagent (0.85 mM CTAB) was added to the cell suspension (30 mg·mL^−1^ in 50 mM PB, pH 7.8). The resulting solution was treated at 30°C in a water bath for 15 min under mixing every 3-4 min. The permeabilized cells were washed by centrifugation (6000 ×g, 5 min) in 10 mM PB, pH 7.8. The precipitated permeabilized cells were resuspended to 30 mg·mL^−1^ in the same buffer solution and stored at +4°C. A half-life of the permeabilized yeast cells was about three weeks of storage at such conditions.

### 2.3. Assay of DLDH Activity in Permeabilized Cells

One unit (1 U) of the* DLDH* activity is defined as that amount of the enzyme which forms 1 *μ*mol hexacyanoferrate(II) per minute under standard conditions of the assay (20°C, 30 mM PB, and pH 7.8). Activity was estimated by spectrophotometric monitoring of hexacyanoferrate(III) reduction at *λ* = 420 nm [19]. During this process, optical density of the analyzed solution becomes lower. Assay mixture consisted of 30 mM PB, рН 7.8; 16 mM sodium D-lactate; 0.83 mM К_3_Fe(CN)_6_; and 20 *μ*L of diluted permeabilized cell suspension (30 mg·mL^−1^).

The specific activity of DLDH was calculated by the following formula:

(1) $$\begin{array}{c} SA=\frac{\Delta E/min\cdot V\cdot n}{EmM\cdot Ccells\cdot Vcells}, \end{array}$$

where Δ*E*/min is change of optical density at *λ* = 420 nm per min; *V* is total volume of the assay solution, mL; *n* is dilution of the enzyme before assay; *E*mM is millimolar extinction of hexacyanoferrate(III), 1.04 mM^−1^·cm^−1^; *V*cells is volume of the added permeabilized cell suspension, mL; and *C*cells is permeabilized cell concentration, mg·mL^−1^.

Specific DLDH activity in the cells was calculated by the following formula, considering difference between specific DLDH activity (+D-lactate) and nonspecific ferrireductase activity (without D-lactate): *A* = *A*_+D-lact_ − *A*_−D-lact_.

## 3. Results and Discussion

The possibility of usage of DLDH-producer as a perspective tool for D-lactate removing from fermented products was investigated. For analysis of D-lactate utilization ability of the recombinant thermotolerant methylotrophic yeast* Hansenula polymorpha* “tr6” (*gcr1 catX/Δcyb2*,* prAOX_DLDH*) constructed by us earlier, overproducing DLDH was chosen. The* H. polymorpha* DLDH-producer was constructed in two steps. First, the gene* CYB2* was deleted on the background of the С-105 (*gcr1*,* catX*) strain of* H. polymorpha* impaired in glucose repression and devoid of catalase activity to avoid specific L-lactate-cytochrome* c* oxidoreductase activity. Second, the homologous gene* DLD1* coding for DLDH was overexpressed under the control of the strong* H. polymorpha* alcohol oxidase promoter in the frame of a plasmid for multicopy integration in the* Δcyb2* strain. The selected recombinant strain possesses 6-fold increased DLDH activity as compared to the initial strain [18]. Two types of yeast cells (intact and permeabilized) were tested. The experiment was carried out as follows: to 50 mL of 10 mM PB, pH 7.8, we added D-lactate (with a final concentration 20 mM) and 0.1 g of the intact yeast cells with specific enzymatic activity 12.6 U·mg^−1^. An incubation of the same amount of permeabilized cells of the* H. polymorpha* “tr6” (with DLDH activity of 11.7 U·mg^−1^) in the same mixture was performed in parallel. The amount of the added yeast cells of two types was calculated related to the specific DLDH activity. The obtained cell mixture was incubated about one hour at a shaker (200 rev.·min^−1^) at 30°C. Every 10 minutes, 2 mL of the mixture was taken, centrifugated (6000 ×g, *r* = 8 cm, 2 min) and the eluates were frozen at −20°C. The residual D-lactate in the frozen samples was analyzed chemically by Buchner method [20] after completing the experiment (Table 1).

**Figure 1:**
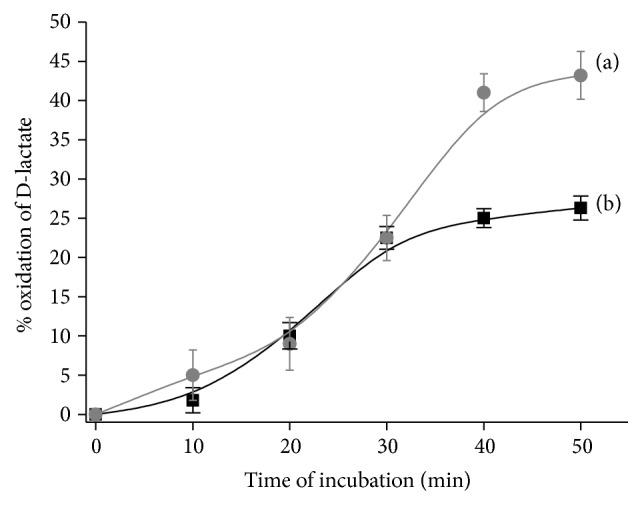
The profile of D-lactate oxidation using recombinant* H. polymorpha* “tr6” cells: (a) permeabilized and (b) living cells. Conditions: 10 mM PB, pH 7.8, 30°C, and shaking (200 rpm) at 30°C; the initial concentration of D-lactate was 20 mM.

The profile of D-lactate utilization using recombinant* H. polymorpha* “tr6” cells is represented in Figure 1.

As shown in Figure 1, effectiveness of D-lactate oxidation (during 40-minute incubation) was calculated as 28% for intact yeast cells (activity of DLDH, 12.6 U·mg^−1^), whereas for the permeabilized cells (with activity of DLDH, 11.2 U·mg^−1^) it was about 41%. The average productivity of the process (for 30 min incubation) was about 9.0 mmol·L^−1^·h^−1^. It was shown that the permeabilized yeast cells are 1.5-fold more effective agent for removal of D-lactate in comparison with intact living cells. Probably this effect could be explained by higher permeability of permeabilized cells to substrate. Thus, for construction of cell-based bioreactor for D-lactate oxidation, permeabilized yeast cells of* H. polymorpha* “tr6” are more effective.

**Figure 2:**
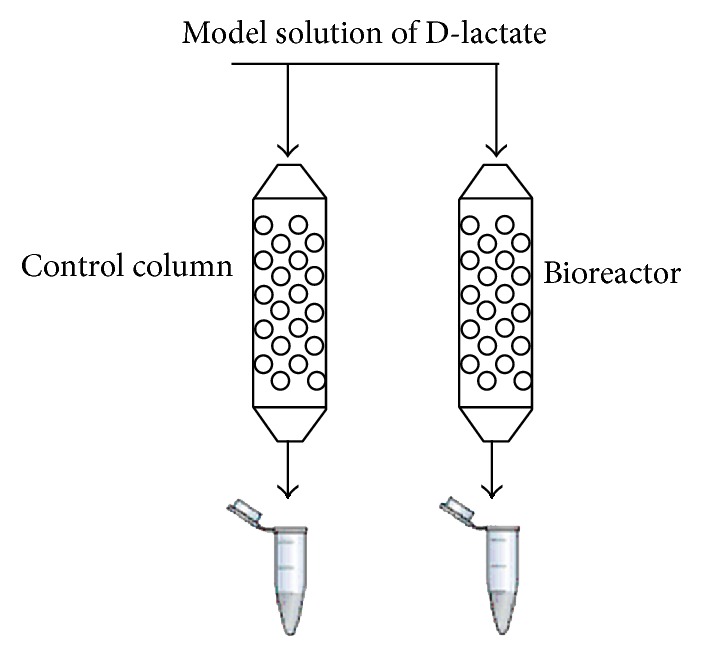
Principal scheme of bioreactor prototype based on permeabilized recombinant yeast cells for removing D-lactate from aqueous solution. The control column contains alginate gel without the cells.

A column-type bioreactor based on permeabilized yeast cells of* H. polymorpha* “tr6” incorporated in alginate gel was constructed. The immobilization of the yeast cells in calcium alginate gel was performed as follows: 2 mL suspension of permeabilized* H. polymorpha* “tr6” cells (60 mg·mL^−1^) was mixed with 2 mL of 4% (w/v) sodium alginate. The obtained mixture was put to medical syringe and dropped through a needle into solution of CaCl_2_ (40 mg·mL^−1^). The formed alginate beads (diameter around 3 mm) with incorporated permeabilized yeast cells were placed to columns 1 × 10 cm (4 mL gel). As a control, “bare” alginate gel without the cells (Figure 2) was used.

The packed columns were washed by 3 mL of PB, pH 7.8, prior to the experiment beginning. As a model solution, 20 mM of D-lactate in 50 mM PB, pH 7.8, containing 1 mM CaCl_2_ was used.

**Figure 3:**
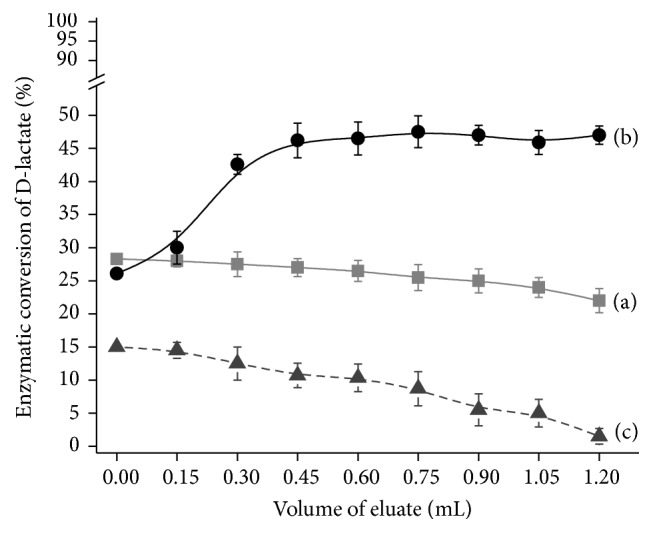
Optimisation of flow rate for enzymatic D-lactate conversion using a column bioreactor, (a) flow rate 50 mL·min^−1^ and (b) flow rate 10 mL·min^−1^, and (c) control column, flow rate 10 mL·min^−1^. Conditions: enzymatic activity of DLDH in permeabilized cells was 80 U·mL^−1^, and the initial D-lactate concentration was 20 mM in the presence of 1 mM CaCl_2_.

For optimisation of flow rate for high efficiency of D-lactate oxidation, the model solution was simultaneously passed through both columns with different speed: 50 mL·min^−1^ and 10 mL·min^−1^. Due to difference in flow rate through the alginate gel the first column with a speed 50 mL·min^−1^ was marked as a “bioreactor 1” and the other one (flow rate 10 mL·min^−1^) was marked as “bioreactor 2,” respectively. Every 15 min, a small amount (0.2 mL) of mixture flowing through the columns was collected and frozen at −20°C. After finishing the experiment, a residual D-lactate in the frozen samples was analyzed by Buhner chemical method of lactate assay (Figure 3).

As was shown in Figure 3, both columns are able to remove D-lactate from the model solution. However, the effectiveness of this process was significantly different and depended on the flow rate through the columns. Thus, in the case of “bioreactor 1” enzymatic conversion of 2 mL 20 mM D-lactate was ranged between 15 and 28%. “Bioreactor 2” showed a twofold higher efficiency of D-lactate oxidation at the same volume of model solution (45–53%). The control alginate column was used to analyze the possibility of nonenzymatic conversion of D-lactate. As was clearly shown in Figure 3, nonenzymatic conversion of D-lactate in control reactor did not occur and the observed decrease in D-lactate concentration (about 14%) was caused by a partial dilution of sample as a result of washing the column by buffer.

The obtained results clearly confirmed the possibility of using permeabilized cells, immobilized in alginate gels, of recombinant DLDH-overproducing strain of* H. polymorpha* “tr6” as a catalyst for bioreactor able to remove D-lactate from fermented food products. It was shown that efficiency of enzymatic conversion of D-lactate for bioreactor prototype tightly depends on the flow rate through the column.

The usage of the developed cell-based prototype for removing toxic D-lactate from fermented products is in progress.

## 4. Conclusions

A laboratory prototype of column-type bioreactor for D-lactate removing from model solution based on permeabilized yeast cells of the* H. polymorpha* “tr6” and alginate gel has been constructed and efficiency of this process has been demonstrated. The optimal concentration of the permeabilized cells (with DLDH activity of 11.2 U·mg^−1^) was estimated as 30 mg per 1 mL of 4% alginate. It was shown that efficiency of enzymatic oxidation of D-lactate by the bioreactor prototype tightly depends on flow rate through the column. The laboratory cell-based bioreactor would be useful in food technology for removing toxic D-lactate.

## Figures and Tables

**Table 1:** The residual D-lactate concentration by Buchner method in the samples selected using two types of the *H. polymorpha *“tr6” cells analyzed.

Time of incubation, min	D-lactate, mМ (permeabilized cells)	D-lactate, mМ (intact cells)
0	20.0 ± 0.00	20.0 ± 0.00
10	19.3 ± 0.15	19.87 ± 0.14
20	15.5 ± 0.2	18.5 ± 0.18
30	11.9 ± 0.23	15.51 ± 0.25
40	11.98 ± 0.28	14.48 ± 0.29
50	12.5 ± 0.30	14.5 ± 0.22
